# Supplementary material for: Role of mobile genetic elements in the global dissemination of the carbapenem resistance gene blaNDM
Source: Nat Commun. 2022 Mar 3;13:1131. doi: 10.1038/s41467-022-28819-2 (PMC8894482; doi:10.1038/s41467-022-28819-2)
Supplement: Supplementary file 1 — Supplementary Information [file 41467_2022_28819_MOESM1_ESM.pdf]

Supplementary Information

Role of mobile genetic elements in the global dissemination of the carbapenem resistance gene *bla*$_{NDM}$

Acman et al.

Supplementary Table 1. NDM-positive samples (and NDM-positive contigs) stratified by where the data was sourced and the associated sequencing platform.

Source/Database		Sequencing Platform: ILLUMINA	ION_TORRENT	PACBIO_SMRT & ILLUMINA (Hybrid assembly)	Unknown
Source/Database	China Hospitals	0	0	104 (105)	0
	Enterobase	185 (185)	0	0	1194 (1194)
	GenBank	0	0	0	1158 (2117)
	RefSeq	0	0	0	2632 (2665)
	SRA	872 (872)	10 (10)	0	0

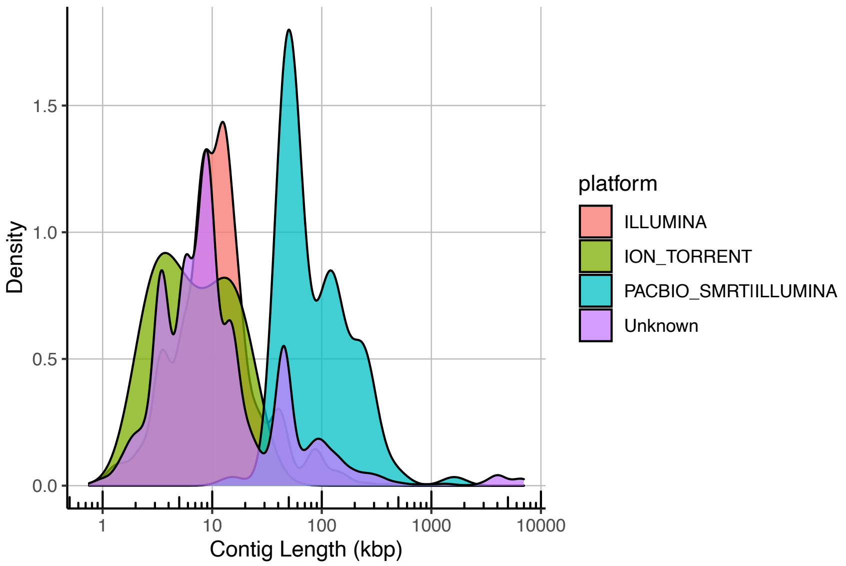

Supplementary Figure 1. Marginal density distribution of the lengths of all assembled *bla*$_{NDM}$-positive contigs depending on the sequencing platform.

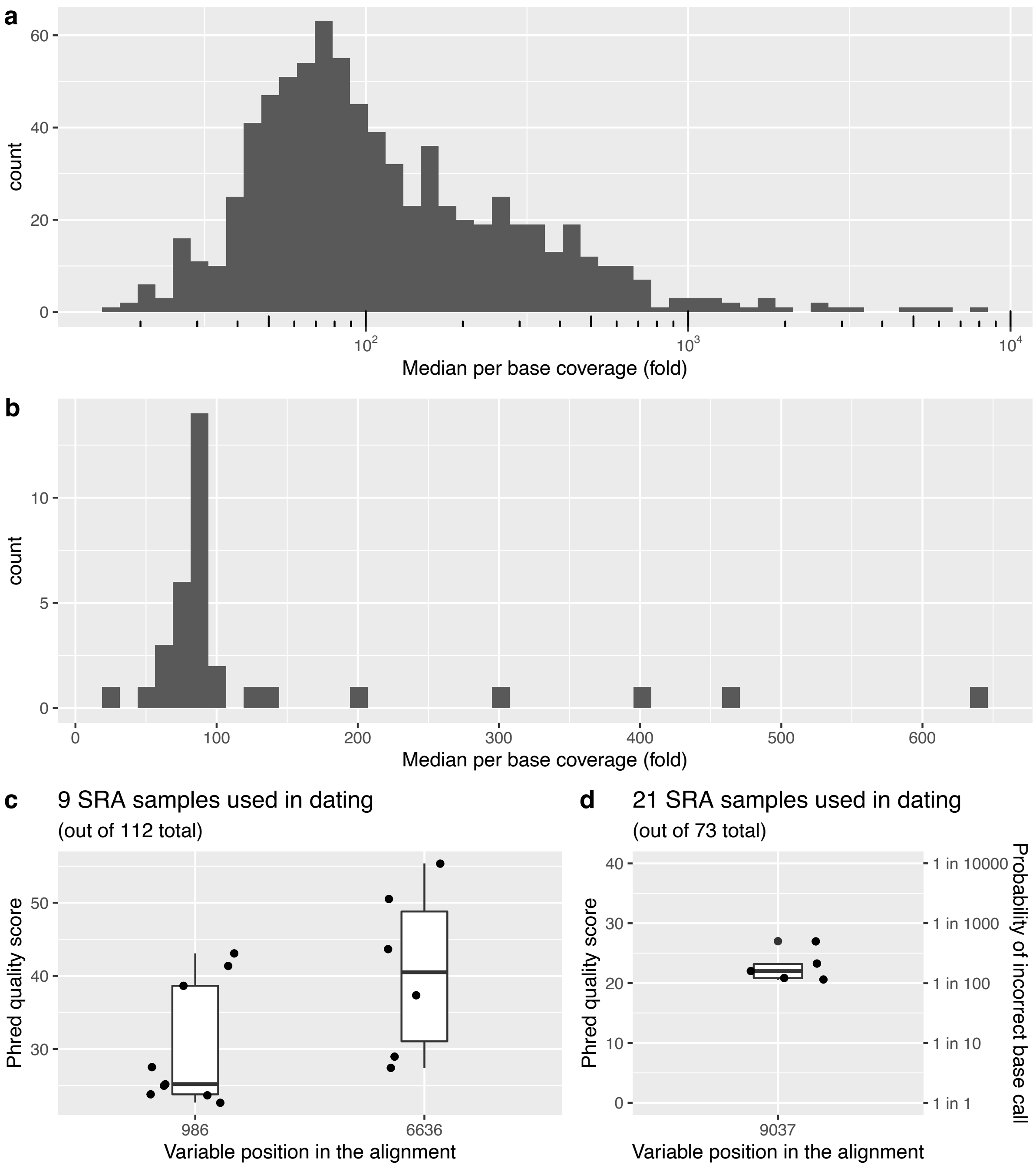

Supplementary Figure 2. Quality assessment of *bla*$_{NDM}$-positive contigs obtained from SRA *de novo* assemblies. Distribution of median per base coverage of *bla*$_{NDM}$-positive contigs is shown for all SRA samples (**a**) and SRA samples used in molecular dating analysis (**b**). Phred quality score per SRA sample (black dot) is shown for all variable positions found in alignments of Tn*125* (n_{986}=9 and n_{6636}=6; **c**) and Tn*3000* (n_{9037}=6; **d**) used in molecular dating. The values presented in the boxplots (**c** and **d**), from left to right respectively, are the following: median values of 25.21, 40.50, and 22.00; lower quartiles of 23.81, 31.0 and 20.84; upper quartiles of 38.65, 48.81, and 23.18; whiskers minima of 22.69, 27.39 and 20.54; and whiskers maxima of 43.08, 55.38 and 23.18.

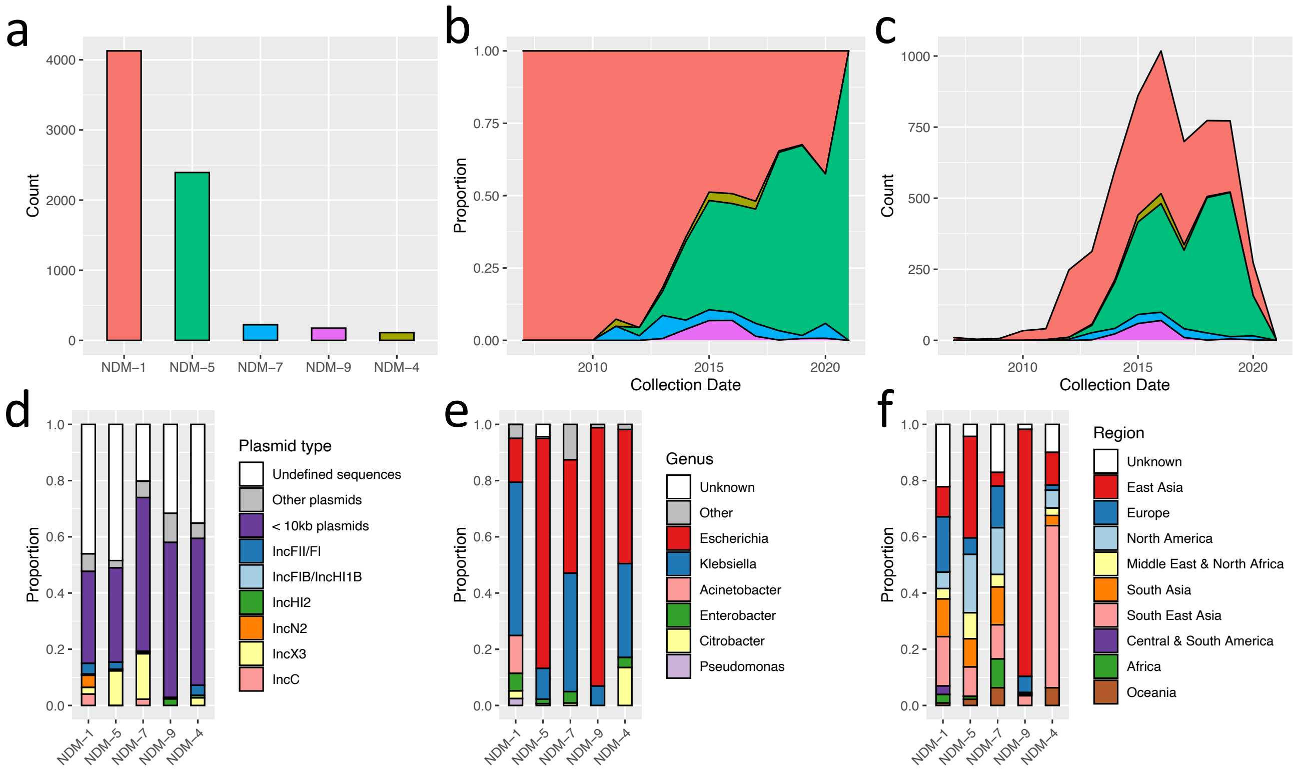

Supplementary Figure 3. Global prevalence and genetic context of NDM variants. Panel (**a**) shows overall counts of the five most frequent NDM variants (i.e., >25 representatives) while panels (**b**) and (**c**) show their prevalence over time. Panels (**d**), (**e**) and (**f**) are bar plots indicating proportions of plasmid backbones, bacterial genera and sampling location respectively across most frequent NDM variants.

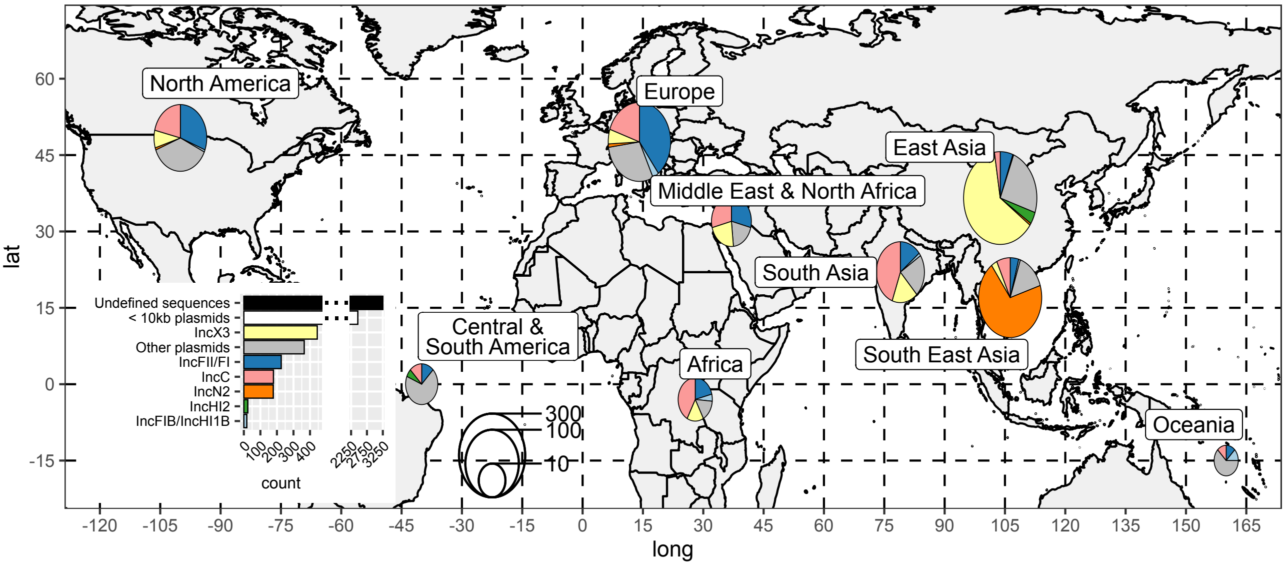

Supplementary Figure 4. Global distribution of plasmid backbones of NDM-positive contigs. All NDM-positive contigs with an identified replicon type were grouped into several broader groups of plasmids. These have been pooled according to the geographical region and represented on the world map using pie charts. The sizes of the pie charts are log-scaled to aid interpretability. The figure inset serves as a legend and indicates counts of plasmid backbones in the dataset. The five largest geographical regions count at minimum 30 different Bioprojects each with majority of samples originating from PRJNA224116, a Refseq Prokaryotic Genome Annotation Project. The world map was rendered from coordinates provided in *rworldmap* package in R.

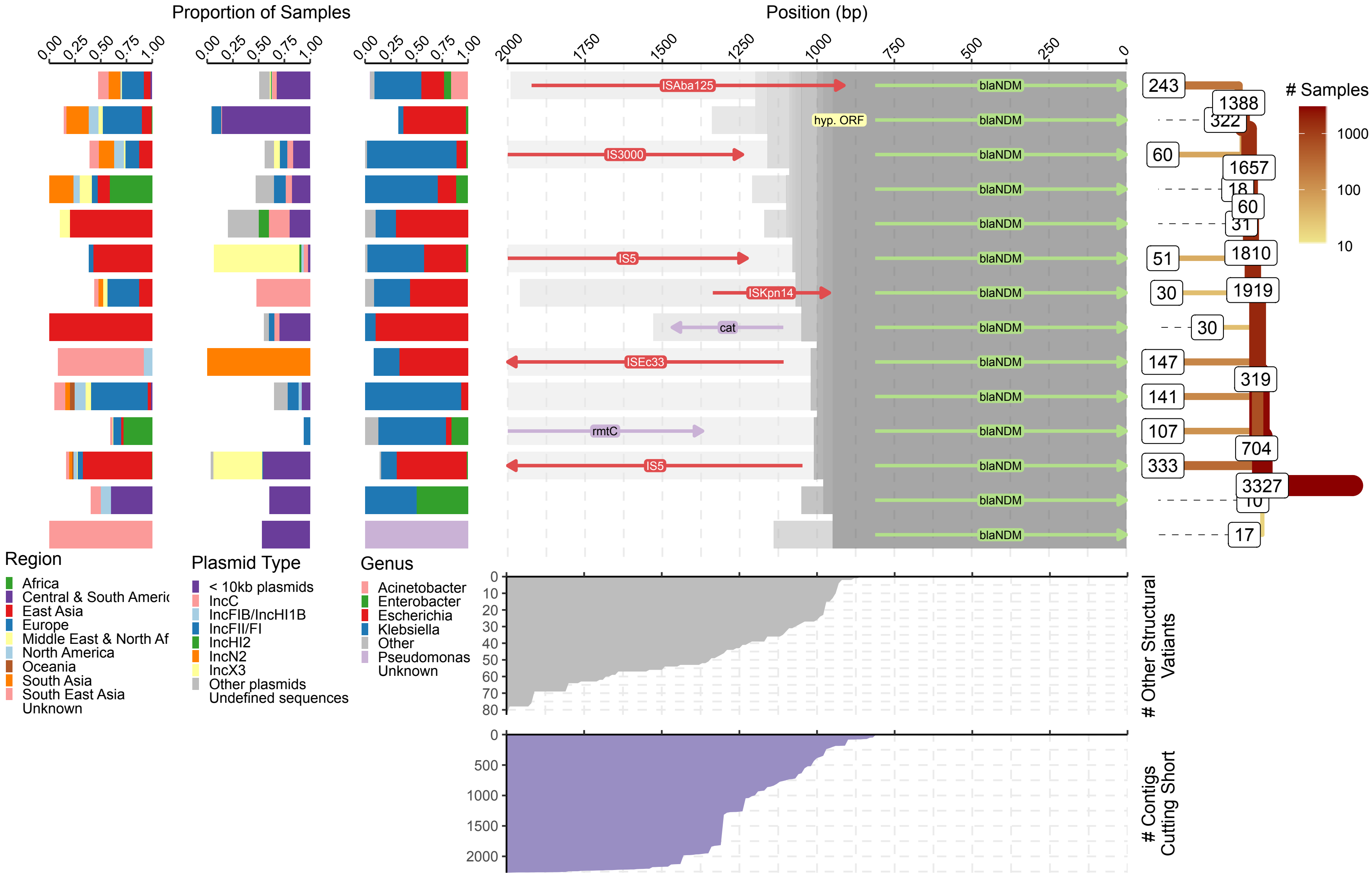
Proportion of Samples
Position (bp)
Samples
Region
Africa
Central & South Americ
East Asia
Europe
Middle East & North Af
North America
Oceania
South Asia
South East Asia
Unknown
Plasmid Type
< 10kb plasmids
IncC
IncFIB/IncHI1B
IncFII/FI
IncHI2
IncN2
IncX3
Other plasmids
Undefined sequences
Genus
Acinetobacter
Enterobacter
Escherichia
Klebsiella
Other
Pseudomonas
Unknown
ISAba125
hyp. ORF
IS3000
IS5
ISKpn14
cat
ISEc33
rmtC
blaNDM
Other Structural Vatiants
Contigs Cutting Short

Supplementary Figure 5. Splitting of structural variants upstream of *bla*NDM. The ‘splitting tree for the most common (i.e., ≥ 10 contigs) structural variants is shown on the right-hand side. The labels on the nodes indicate the number of contigs remaining on each branch. The other contigs either belong to other structural variants or were removed due to being too short in length. The number of contigs cutting short is indicated by the area chart at the bottom. Similarly, the number of less common structural variants is indicated by the upper area chart. The genome annotations provided by the Prokka and Roary pipelines of the most common structural variants are shown in the middle of the figure. The homologous regions across structural variants are indicated by the grey shading. Some of the structural variants and branches were intentionally cut short even though their contigs were of sufficient size. This was done in order to prevent excessive bifurcation and to make the tree easier to interpret. Branches with more than 75% of contigs lost due to variation and short length were truncated. The distribution of genera, plasmid types and geographical regions of samples that belong to a each of the common structural variant is shown on the left-hand side.

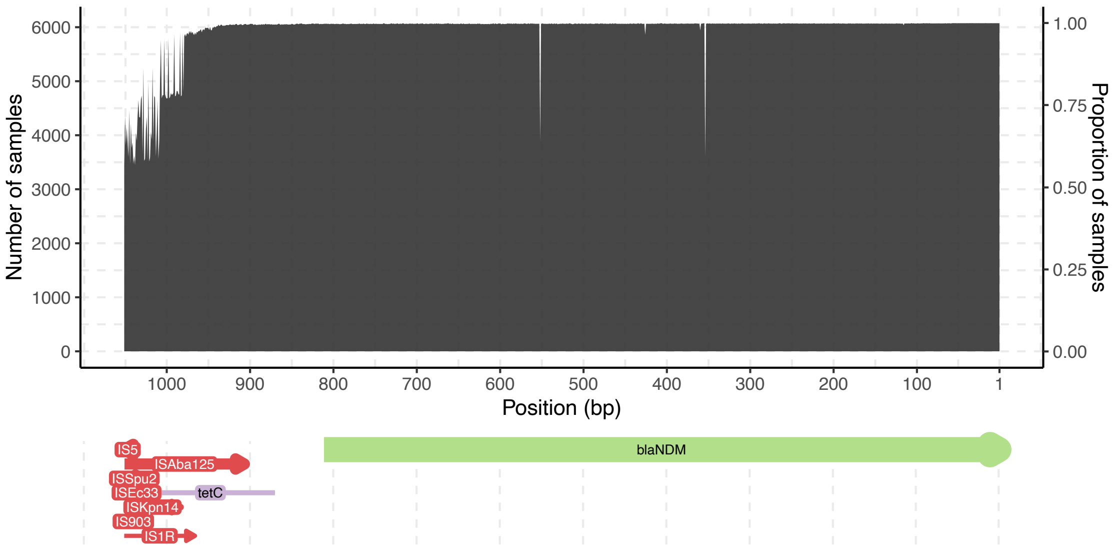

Supplementary Figure 6. Alignment of 6455 sufficiently long contigs 1050bp upstream of *bla*NDM stop codon.

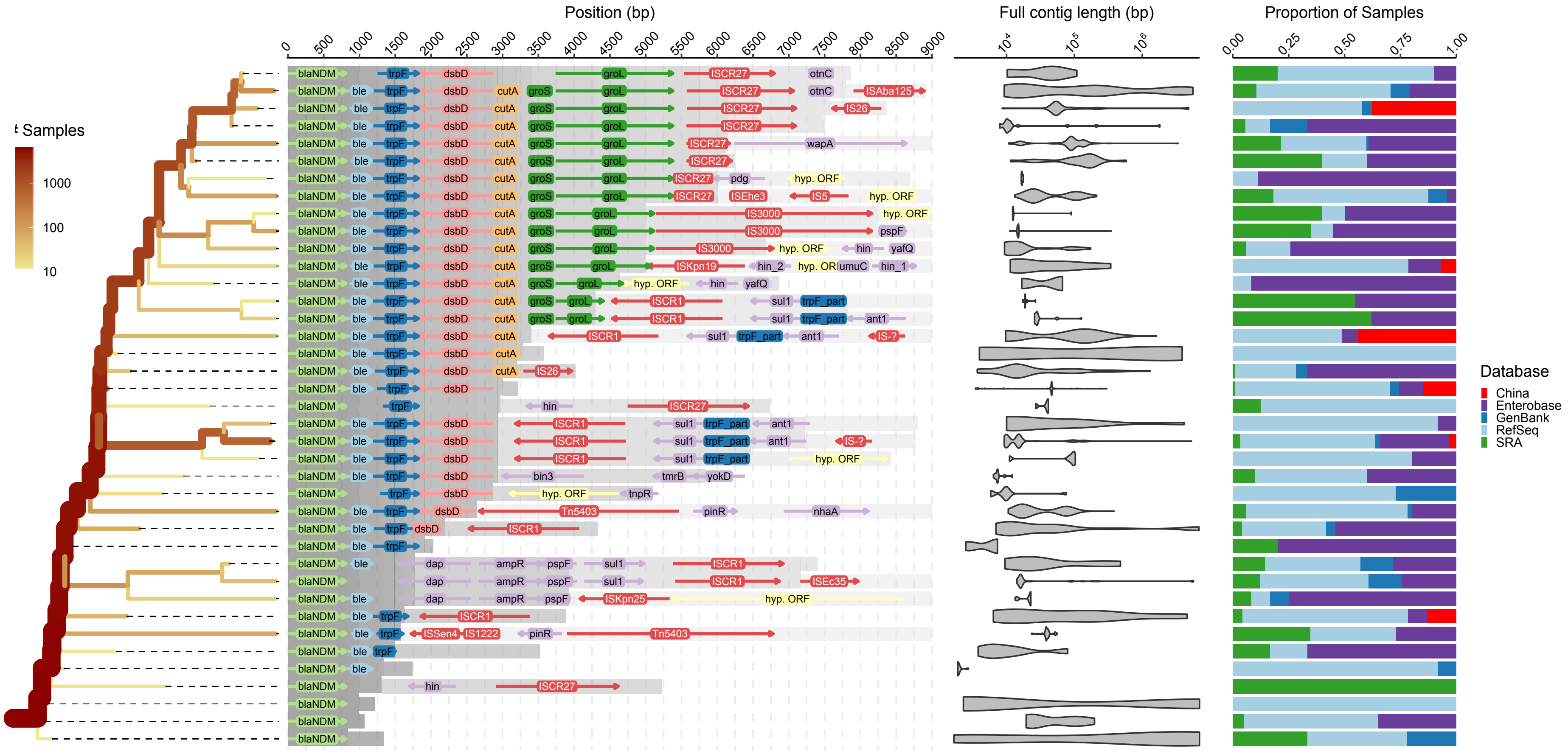

Supplementary Figure 7. Splitting of structural variants upstream of *bla*$_{\mathrm{NDM}}$. This is an extension of Figure 3. The thickness and the hue of the branches reflect the number of contigs on each branch. The genome annotations of the most common structural variants are shown in the middle of the figure and the grey shading indicates homology across structural variants. To aid interpretability, some of the structural variants and branches were intentionally cut short. The distributions of contig lengths and source databases are shown on the left-hand side.

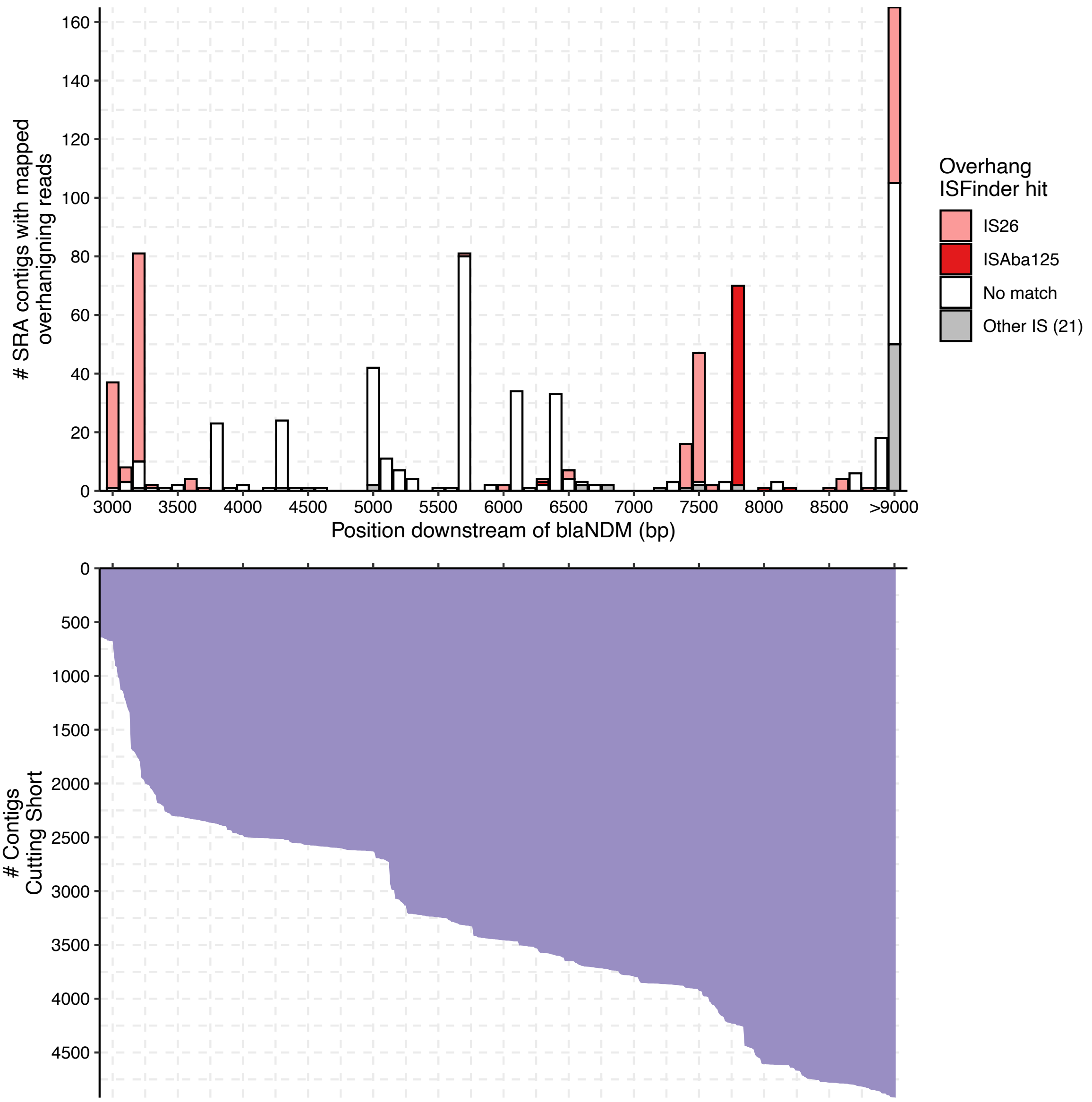

Supplementary Figure 8. Mapping of overhangs of *bla*NDM-carrying contigs to the ISFinder database. Reads of 781 Illumina paired-end sequencing datasets from SRA was mapped back to the corresponding contigs with length downstream of *bla*NDM ≥3000bp. Downstream overhangs ≥50bp were then screened against ISFinder database. Top panel represents the distribution of ISFinder hits over the lengths of contigs downstream of *bla*NDM start codon. The bottom panel is an excerpt from Figure 3 and shows a cumulative distribution of all contig lengths downstream of *bla*NDM start codon. From the bottom panel, three sharp increases in number of short contig can be distinguished at positions: 3000-3300 bp, 5000-5250 bp, and 7500-8000 bp.

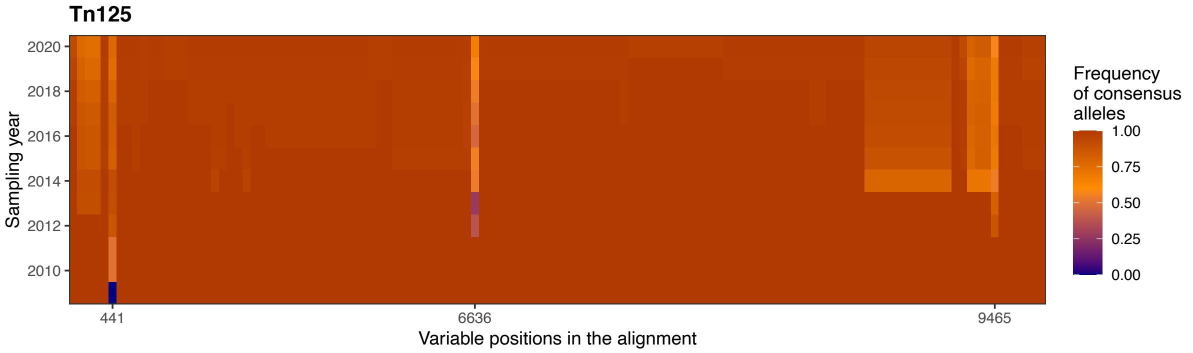

Position: 441 (consensus: a)

year	a	t	g	c	–	n_samples
2009	0	0	0	1	0	1
2010	0.5	0	0	0.5	0	2
2011	0.5	0	0	0.5	0	2
2012	0.88	0	0	0.12	0	8
2013	0.91	0	0	0.09	0	11
2014	0.92	0	0	0.08	0	24
2015	0.82	0	0	0.18	0	45
2016	0.84	0	0	0.16	0	61
2017	0.84	0	0	0.16	0	68
2018	0.8	0	0	0.2	0	74
2019	0.76	0	0	0.24	0	86
2020	0.78	0	0	0.22	0	108

Position: 6636 (consensus: g)

year	a	t	g	c	–	n_samples
2009	0	0	1	0	0	1
2010	0	0	1	0	0	2
2011	0	0	1	0	0	2
2012	0.62	0	0.38	0	0	8
2013	0.73	0	0.27	0	0	11
2014	0.46	0	0.54	0	0	24
2015	0.44	0	0.56	0	0	45
2016	0.54	0	0.46	0	0	61
2017	0.5	0	0.5	0	0	68
2018	0.46	0	0.54	0	0	74
2019	0.42	0	0.58	0	0	86
2020	0.33	0	0.67	0	0	108

Position: 9465 (consensus: c)

year	a	t	g	c	–	n_samples
2009	0	0	0	1	0	1
2010	0	0	0	1	0	2
2011	0	0	0	1	0	2
2012	0.12	0	0	0.88	0	8
2013	0.18	0	0	0.82	0	11
2014	0.46	0	0	0.54	0	24
2015	0.31	0	0	0.69	0	45
2016	0.3	0	0	0.7	0	61
2017	0.32	0	0	0.68	0	68
2018	0.31	0	0	0.69	0	74
2019	0.31	0	0	0.69	0	86
2020	0.43	0	0	0.57	0	108

Supplementary Figure 9. Temporal patterns across variable positions in the alignment of the *Tn*125 transposon. A heatmap shows the frequency of consensus sequence alleles in *Tn*125 alignment over time. Allele frequency tables of more variable positions are given below the heatmap.

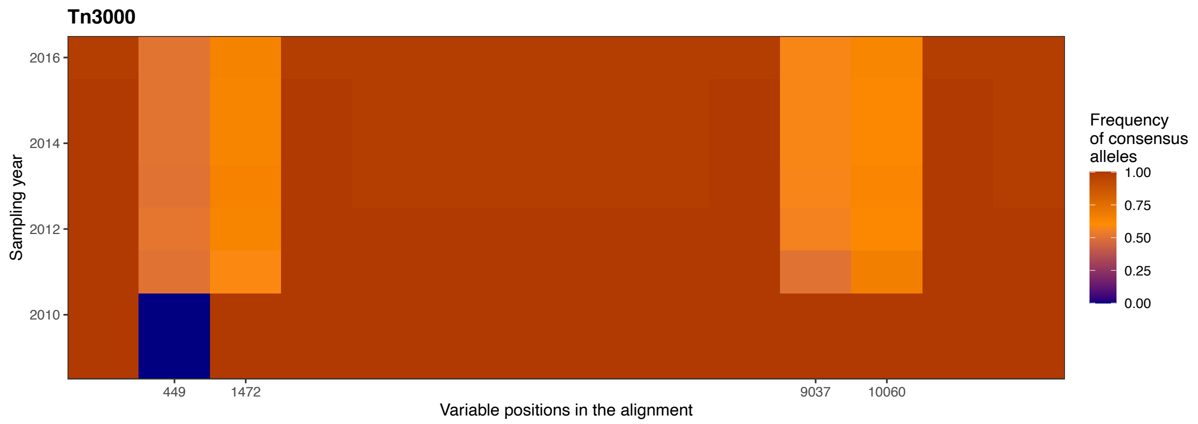

Position: 449 (consensus: a)

year	a	t	g	c	–	n_samples
2009	0	1	0	0	0	1
2010	0	1	0	0	0	1
2011	0.5	0.5	0	0	0	12
2012	0.52	0.48	0	0	0	66
2013	0.5	0.5	0	0	0	68
2014	0.51	0.49	0	0	0	71
2015	0.51	0.49	0	0	0	71
2016	0.51	0.49	0	0	0	73

Position: 1472 (consensus: a)

year	a	t	g	c	–	n_samples
2009	1	0	0	0	0	1
2010	1	0	0	0	0	1
2011	0.58	0.42	0	0	0	12
2012	0.64	0.36	0	0	0	66
2013	0.65	0.35	0	0	0	68
2014	0.63	0.37	0	0	0	71
2015	0.63	0.37	0	0	0	71
2016	0.64	0.36	0	0	0	73

Position: 9037 (consensus: t)

year	a	t	g	c	–	n_samples
2009	0	1	0	0	0	1
2010	0	1	0	0	0	1
2011	0.5	0.5	0	0	0	12
2012	0.44	0.56	0	0	0	66
2013	0.43	0.57	0	0	0	68
2014	0.42	0.58	0	0	0	71
2015	0.42	0.58	0	0	0	71
2016	0.42	0.58	0	0	0	73

Position: 10060 (consensus: a)

year	a	t	g	c	–	n_samples
2009	1	0	0	0	0	1
2010	1	0	0	0	0	1
2011	0.67	0.33	0	0	0	12
2012	0.62	0.38	0	0	0	66
2013	0.63	0.37	0	0	0	68
2014	0.62	0.38	0	0	0	71
2015	0.62	0.38	0	0	0	71
2016	0.63	0.37	0	0	0	73

Supplementary Figure 10. Temporal patterns across variable positions in the alignment of the *Tn*3000 transposon. A heatmap shows the frequency of consensus sequence alleles in *Tn*3000 alignment over time. Allele frequency tables of more variable positions are given below the heatmap.

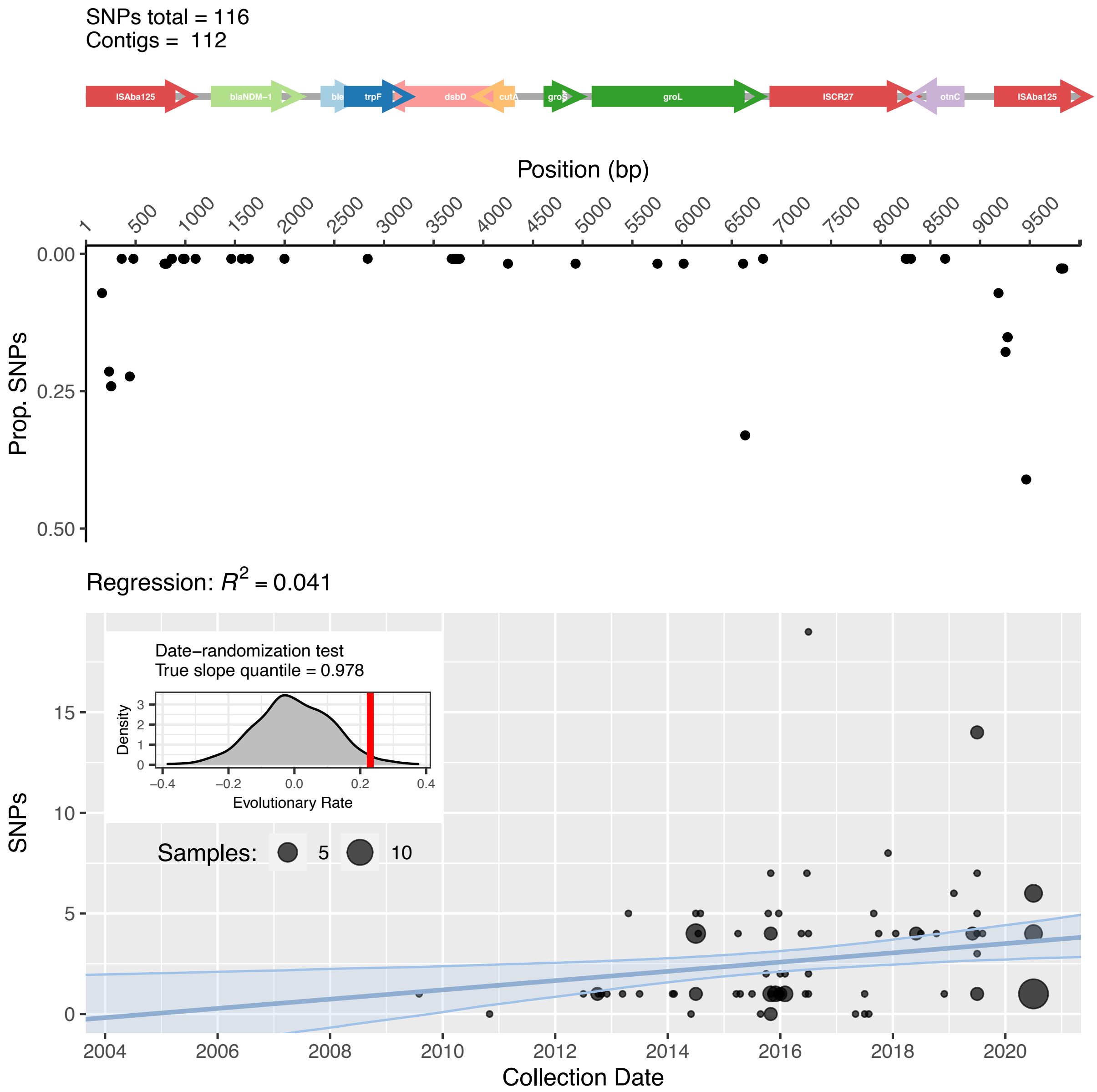

Supplementary Figure 11. Assessment of temporal signal in the alignment of *Tn*125. Starting from the top, the figure contains the following plots: schematic representation of the aligned transposon sequences; the SNP frequency across the alignment evaluated against the inferred ancestral sequence; the linear regression analysis of number of SNPs accumulated against the year of sample collection. The ribbon surrounding the regression line provides a 95% confidence interval given by the bootstrapping the regression analysis (1000 iterations). The regression line is defined by the function: y=x*0.2302251 – 461.5534. The inset of the regression plot shows the results of the date-randomization test. The marginal distribution of the inset indicates the regression line slope values (i.e., evolutionary rates) after 1000 date randomizations and the red vertical line indicates the true slope value.

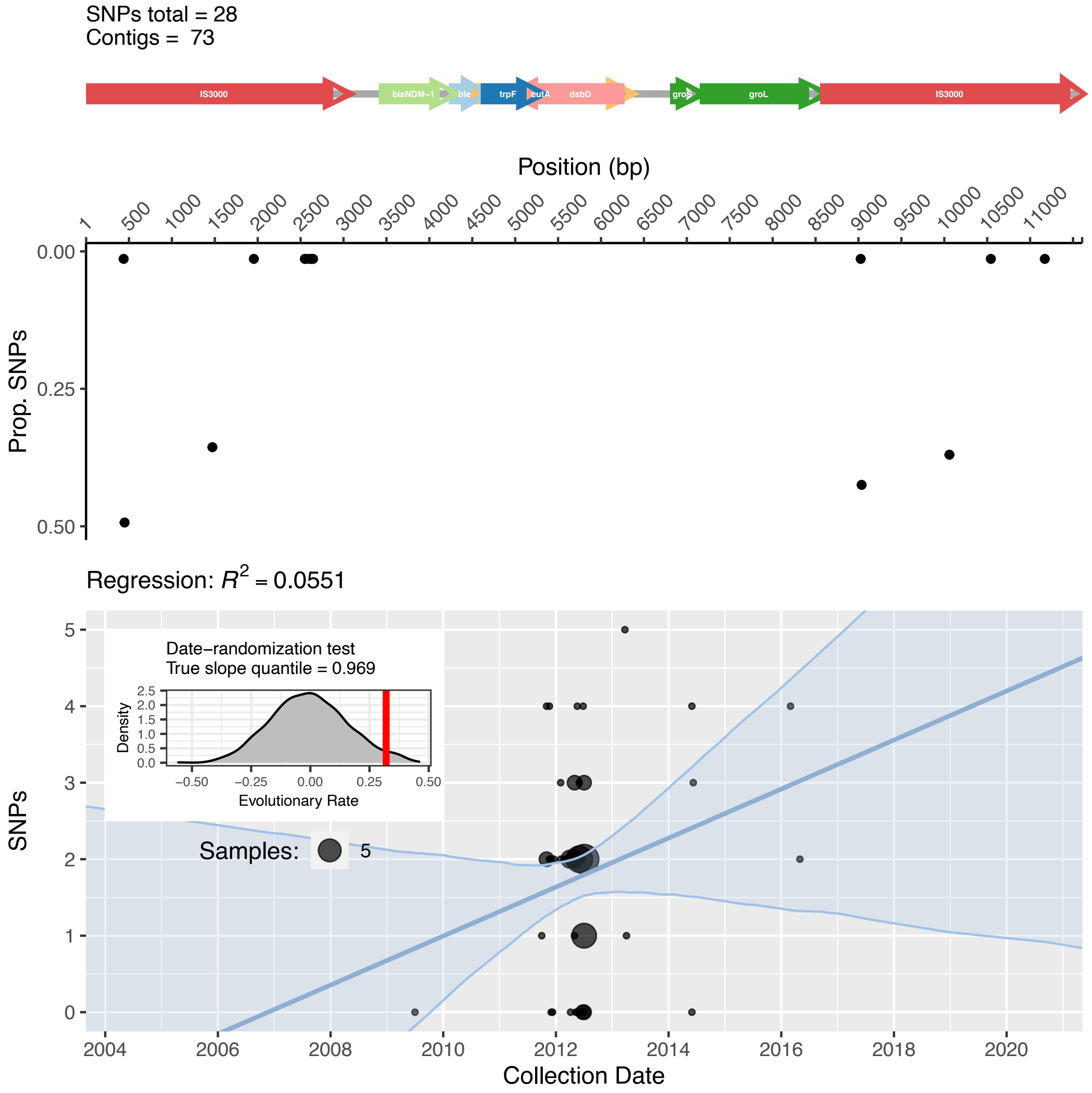

Supplementary Figure 12. Assessment of temporal signal in the alignment of *Tn*3000. Starting from the top, the figure contains the following plots: schematic representation of the aligned transposon sequences; the SNP frequency across the alignment evaluated against the inferred ancestral sequence; the linear regression analysis of number of SNPs accumulated against the year of sample collection. The ribbon surrounding the regression line provides a 95% confidence interval given by the bootstrapping the regression analysis (1000 iterations). The regression line is defined by the function: y=x*0.3202451 – 642.697. The inset of the regression plot shows the results of the date-randomization test. The marginal distribution of the inset indicates the regression line slope values (i.e., evolutionary rates) after 1000 date randomizations and the red vertical line indicates the true slope value.

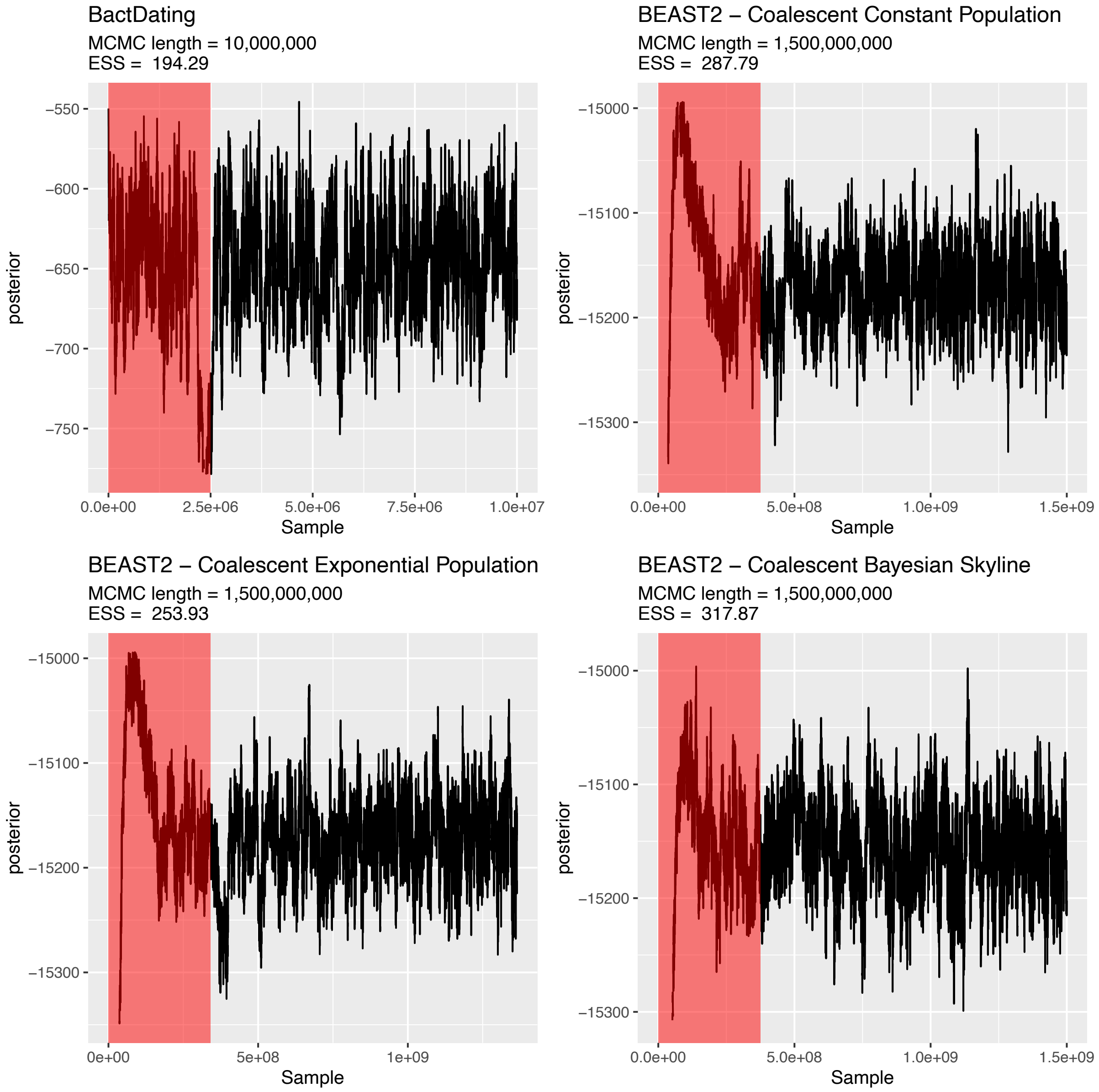

Supplementary Figure 13. MCMC trace plots of the posterior for four Bayesian molecular tip-dating analyses of *Tn*125. The MCMC lengths and effective sample sizes (ESS; evaluated using *coda* R package) are provided above each panel. The red shading indicates 20% of burn-in.

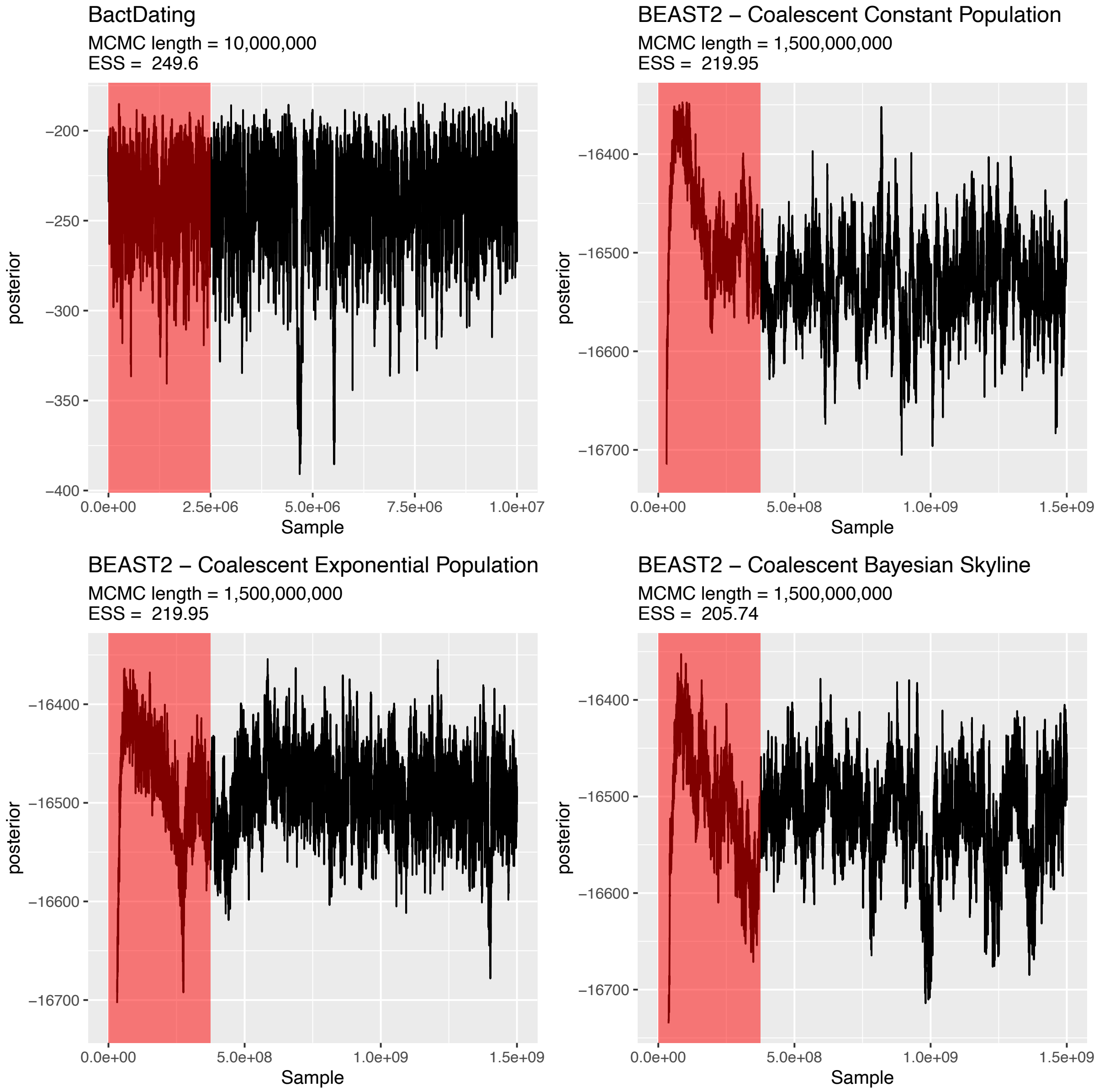

Supplementary Figure 14. MCMC trace plots of the posterior for four Bayesian molecular tip-dating analyses of *Tn*3000. The MCMC lengths and effective sample sizes (ESS; evaluated using *coda* R package) are provided above each panel. The red shading indicates 20% of burn-in.

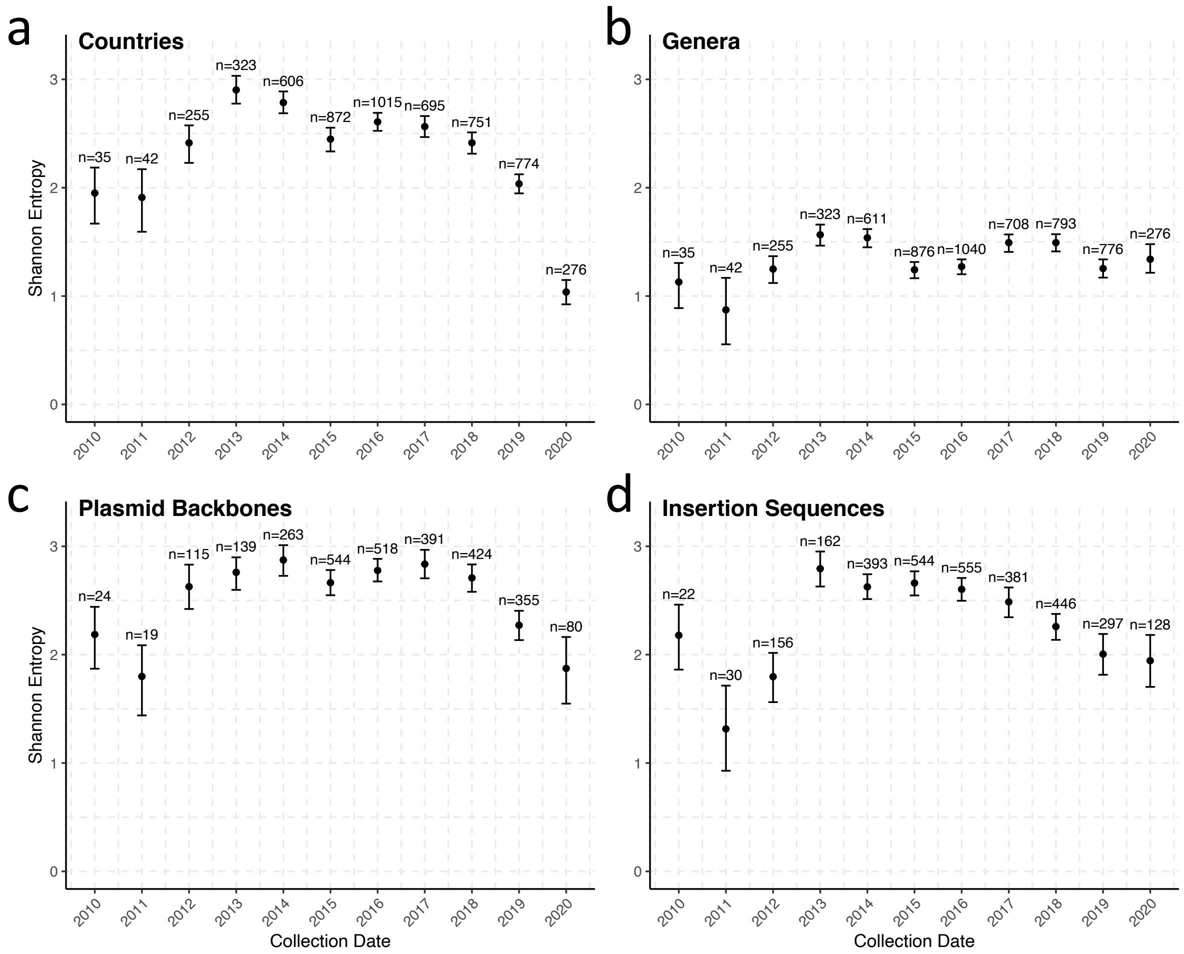

Supplementary Figure 15. Change in Shannon entropy (diversity) over time for four categories of NDM-positive samples. More specifically, the entropy was estimated for samples' country labelling (**a**), bacterial genera (**b**), plasmid backbones bearing *bla*$_{NDM}$ gene (as determined by mapping to plasmid reference sequences, **c**), Insertion sequences found in the vicinity (≤ 10Kb) of *bla*$_{NDM}$ gene (**d**). The median entropy (points) together with 95% confidence interval (error bars) was estimated using bootstrapping with replacement (1000 iterations). Years with too few samples (≤10) were excluded from the analysis.

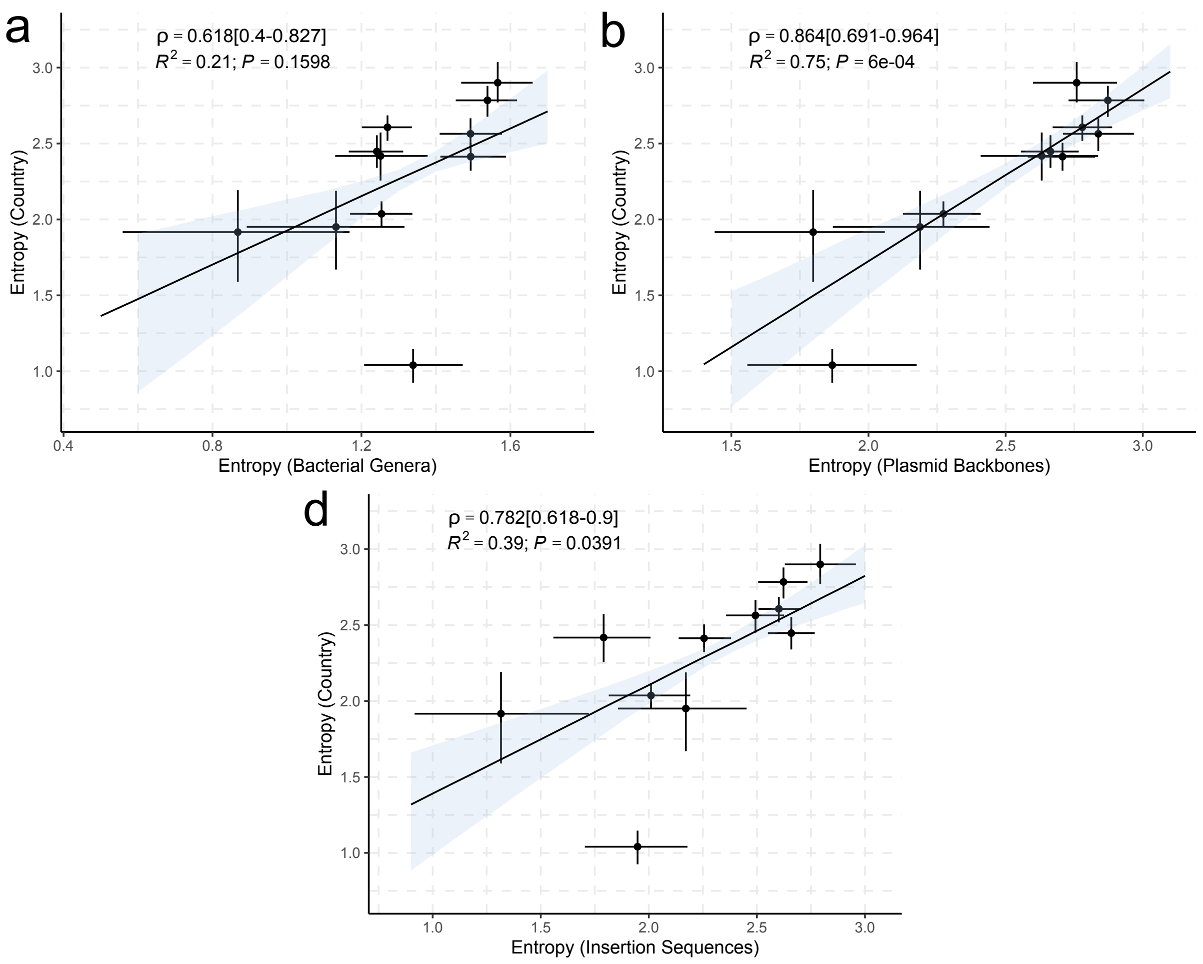

Supplementary Figure 16. Spearman correlation and linear regression between Shannon entropy (diversity) estimates. The Shannon entropy bootstrapped values (Supplementary Figure 15) were used to provide a median and 95% confidence interval (CI) of Spearman correlation coefficients, as well as a median regression line with 95% CI (ribbon) between samples' country labelling and: bacterial genera (**a**), plasmid backbones (**b**), and Insertion Sequences (**c**). The sample size (n) used for calculating each confidence interval is given in Supplementary Figure 15 above each data point. F-test was used in regression analysis to determine statistical significance of the slope coeficient. No adjustments were made for multiple testing.

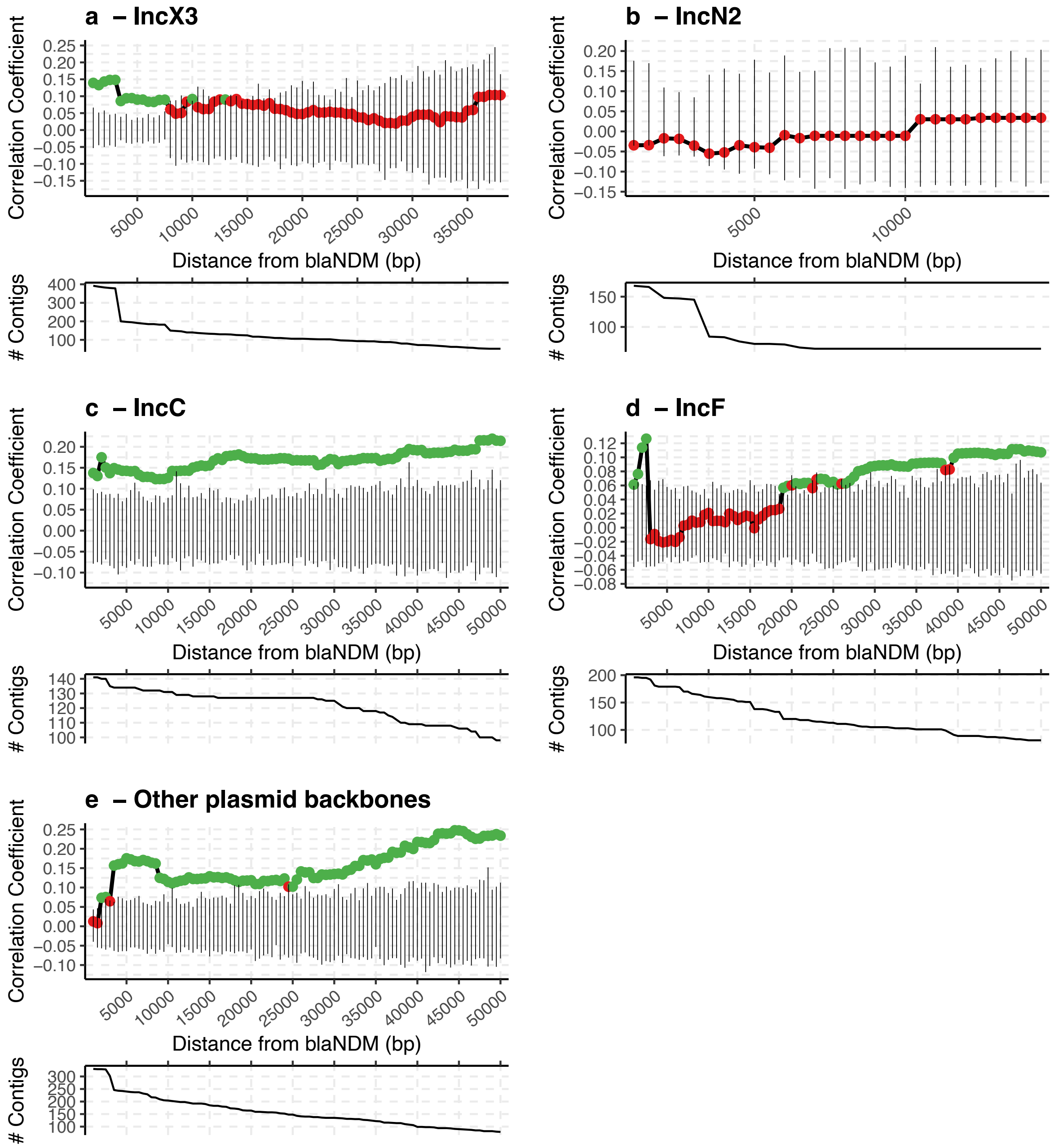

Supplementary Figure 17. The spearman correlation estimates between genetic and geographic distance of NDM-positive contigs as the DNA sequence upon which the genetic distance is measured is increased downstream of *bla*$_{\mathbf{NDM}}$ gene. The exact Jaccard Distance (JD), an alignment-free metric, was used as a measure of genetic distance. Geographic distance between samples was estimated by the *geodist* (v0.0.6) R package using sampling coordinates. The analysis was performed on contigs with confirmed IncX3 (**a**), IncN2 (**b**), IncC (**c**), and IncF (**d**) replicon types, and other plasmid backbones >10 Kb (**e**). The genetic and geographic distance was measured between all pairs of contigs which yielded two distance matrices: genetic and geographic. The Spearman correlation was then estimated between two matrices and its significance evaluated using Mantel (randomization) test. Significant Spearman correlations (p-value <0.05) are indicated with green points and non-significant correlations with the red point, while the black vertical lines provide the 95% confidence interval of 100

Mantel test permutations. The genetic distance matrix and subsequent Spearman correlation were estimated multiple times by increasing the assessed DNA sequence starting from *bla*$_{NDM}$ gene and continuing downstream. The plot below each correlation graph indicates the number of contigs used in the correlation analysis as the assessed DNA sequence is increased.
