# Supplementary material for: Role of mobile genetic elements in the global dissemination of the carbapenem resistance gene blaNDM
Source: Nat Commun. 2022 Mar 3;13:1131. doi: 10.1038/s41467-022-28819-2 (PMC8894482; doi:10.1038/s41467-022-28819-2)
Supplement: Supplementary file 2 — Reporting Summary [file 41467_2022_28819_MOESM2_ESM.pdf]

nature portfolio

Corresponding author(s): Mislav Acman

Last updated by author(s): Feb 10, 2022

Reporting Summary

Nature Portfolio wishes to improve the reproducibility of the work that we publish. This form provides structure for consistency and transparency in reporting. For further information on Nature Portfolio policies, see our Editorial Policies and the Editorial Policy Checklist.

Statistics

For all statistical analyses, confirm that the following items are present in the figure legend, table legend, main text, or Methods section.

n/a	Confirmed	
☐	☒	The exact sample size (*n*) for each experimental group/condition, given as a discrete number and unit of measurement
☐	☒	A statement on whether measurements were taken from distinct samples or whether the same sample was measured repeatedly
☐	☒	The statistical test(s) used AND whether they are one- or two-sided<br>*Only common tests should be described solely by name; describe more complex techniques in the Methods section.*
☐	☒	A description of all covariates tested
☐	☒	A description of any assumptions or corrections, such as tests of normality and adjustment for multiple comparisons
☐	☒	A full description of the statistical parameters including central tendency (e.g. means) or other basic estimates (e.g. regression coefficient) AND variation (e.g. standard deviation) or associated estimates of uncertainty (e.g. confidence intervals)
☐	☒	For null hypothesis testing, the test statistic (e.g. *F*, *t*, *r*) with confidence intervals, effect sizes, degrees of freedom and *P* value noted<br>*Give P values as exact values whenever suitable.*
☐	☒	For Bayesian analysis, information on the choice of priors and Markov chain Monte Carlo settings
☒	☐	For hierarchical and complex designs, identification of the appropriate level for tests and full reporting of outcomes
☐	☒	Estimates of effect sizes (e.g. Cohen's *d*, Pearson's *r*), indicating how they were calculated

Our web collection on statistics for biologists contains articles on many of the points above.

Software and code

Policy information about availability of computer code

Data collection	Raw sequencing reads were assembled using Unicycler (v0.4.8) or SPAdes (v3.11.1) software. EnteroBase was screened using BlastFrost (v1.0.0). Bitsliced Genomic Signature Index (BIGSI) tool (v0.3) was used to identify all Sequence Read Archive (SRA) unassembled reads which carry the blaNDM gene.
Data analysis	The following software have been used in data analysis: BLAST (v2.10.1+) -- database screenings, plasmid annotation and pairwise contig comparisons; taxize (v0.9.99) -- R package used for retrieving information on taxonomy; Geocoding API from Google (v3.44.9) -- used for retrieving coordinates based on sampling location names; Prokka (v1.14.6) and Roary (v3.13.0) -- annotation of NDM positive contigs; Mash (v2.2) -- screening of the plasmid database; Cytoscape (v3.8.0) -- network visualization; igraph (1.2.11)-- R package used in network analysis; BBMap (v38.59) -- analysis of overhanging reads; Clustal Omega (v1.2.3) -- used for building multiple sequence alignments used in molecular dating; UGENE (v38.0) -- inspection and manual proofing of the multiple sequence alignments; RAxML (v8.2.12) -- used for building a tree prior used in molecular dating analysis; BEAST2 (v2.6.0) and BactDating (v1.0.12)-- molecular dating; coda (v0.194) -- R package used for calculating effective sample sizes (ESS) after molecular dating analysis; Bindash (v0.2.1) -- used for calculating alignment free genetic distance between NDM positive contigs; geodist (v0.0.7) -- R package used for estimating geographical distance between sampling locations. Aditional scripts used for tracking structural variants around blaNDM genes are available on GitHub: https://github.com/macman123/track_structural_variants

For manuscripts utilizing custom algorithms or software that are central to the research but not yet described in published literature, software must be made available to editors and reviewers. We strongly encourage code deposition in a community repository (e.g. GitHub). See the Nature Portfolio guidelines for submitting code & software for further information.

Data

Policy information about availability of data

All manuscripts must include a data availability statement. This statement should provide the following information, where applicable:

- Accession codes, unique identifiers, or web links for publicly available datasets
- A description of any restrictions on data availability
- For clinical datasets or third party data, please ensure that the statement adheres to our policy

Sequence data of samples carrying blaNDM gene was collected from the following databases: NCBI RefSeq, NCBI's GenBank, EnteroBase, and NCBI Sequence Read Archive (SRA). Assembled genomes of 104 newly sequenced bacterial samples from Chinese hospitals and animal farms are available on SRA and GenBank, respectively, under under the accession number: PRJNA761884. Complete metadata containing the accession numbers of bacterial genomes used in our analyses can be found in Supplementary Data 1 file. Filtered dataset of 7,148 NDM positive contigs used in our analyses is available on Figshare under the following DOI: 10.5522/04/16594784. Accession codes of the complete plasmids used in the analysis is provided in Supplementary Data 2.

Field-specific reporting

Please select the one below that is the best fit for your research. If you are not sure, read the appropriate sections before making your selection.

☒ Life sciences ☐ Behavioural & social sciences ☐ Ecological, evolutionary & environmental sciences

For a reference copy of the document with all sections, see nature.com/documents/nr-reporting-summary-flat.pdf

Life sciences study design

All studies must disclose on these points even when the disclosure is negative.

Sample size	Overall, the dataset (post filtering) contained 6,155 bacterial genomes carrying the blaNDM gene. The filtered dataset of contigs used to determine structural variations of around blaNDM gene, in molecular dating, and in all other analyses was comprised of 7,148 blaNDM-carrying contigs. To the best of our knowledge, this dataset represents all available blaNDM positive sequences at the time the analysis commenced.
Data exclusions	Following the initial collection and assembly of genomes from various sources, the following filtering of the data was applied:<br>--> 48 contigs were found to carry more than one copy of blaNDM and were not included in the final dataset<br>--> 88 contigs were found to have a partial blaNDM sequence (<90% length coverage) and were not included in the final dataset<br>--> 14 assemblies had a single blaNDM gene split into two contigs (likely due to an insertion) and were removed<br>--> several contigs were removed due to poor assembly quality which was manually assessed.
Replication	To ensure reproducibility of the results, all accession codes and the metadata used in the analyses was provided in Supplementary Data 1. In addition, filtered dataset is provided on Figshare, and newly sequenced data from China is deposited on SRA and GenBank. BEAST2 xml configuration files used for the dating analysis are provided as Supplementary Data 3. An implementation of the algorithm used to track structural variations around blaNDM gene is available on Github. All other methods and software are described in Methods and Results section. All of the results presented here are obtained using various statistical methods and data analysis. Provided one uses the exact dataset, all the results should be absolutely reproducible.
Randomization	The randomization is not applicable due to design of this study. No random assignment of samples was performed prior to measurements (i.e. sequencing) being taken.
Blinding	The blinding is not applicable due to design of this study. No information could be hidden that would alter the outcome of this study.

Reporting for specific materials, systems and methods

We require information from authors about some types of materials, experimental systems and methods used in many studies. Here, indicate whether each material, system or method listed is relevant to your study. If you are not sure if a list item applies to your research, read the appropriate section before selecting a response.

Materials & experimental systems

n/a	Involved in the study
☒	☐ Antibodies
☒	☐ Eukaryotic cell lines
☒	☐ Palaeontology and archaeology
☒	☐ Animals and other organisms
☒	☐ Human research participants
☒	☐ Clinical data
☒	☐ Dual use research of concern

Methods

n/a	Involved in the study
☒	☐ ChIP-seq
☒	☐ Flow cytometry
☒	☐ MRI-based neuroimaging
