# Supplementary material for: Role of mobile genetic elements in the global dissemination of the carbapenem resistance gene blaNDM
Source: Nat Commun. 2022 Mar 3;13:1131. doi: 10.1038/s41467-022-28819-2 (PMC8894482; doi:10.1038/s41467-022-28819-2)
Supplement: Supplementary file 4 — Description of Additional Supplementary Files [file 41467_2022_28819_MOESM4_ESM.pdf]

Title: Supplementary Data 1.
Description: A table containing information about the blaNDM positive contigs analysed in this work. This includes corresponding sample accession numbers and other relevant information: instrument, organism, collection date, sampling location, sampling source, identified plasmid types and plasmid cluster, length, and GC content.

Title: Supplementary Data 2.
Description: A table containing information about the dataset of complete plasmid reference sequences. This includes accession numbers of complete plasmids, description, plasmid cluster, length, organism, collection date, isolation source and sampling location.

Title: Supplementary Data 3.
Description: XML configuration files used in BEAST2 molecular dating analysis.
